# The Effect of Vascular Endothelial Growth Factor on Osteoclastogenesis in Rheumatoid Arthritis

**DOI:** 10.1371/journal.pone.0124909

**Published:** 2015-04-20

**Authors:** Hae-Rim Kim, Kyoung-Woon Kim, Bo-Mi Kim, Mi-La Cho, Sang-Heon Lee

**Affiliations:** 1 Department of Rheumatology, Research Institute of Medical Science, Konkuk University School of Medicine, Seoul, Korea; 2 Conversant Research Consortium in Immunologic disease, Seoul St. Mary's Hospital, College of Medicine, The Catholic University of Korea, Seoul, Korea; 3 The Rheumatism Research Center, Catholic Research Institute of Medical Science, The Catholic University of Korea, Seoul, Korea; China Medical University, TAIWAN

## Abstract

Vascular endothelial growth factor (VEGF) has angiogenic, inflammatory, and bone-destructive roles in rheumatoid arthritis (RA). We aimed to determine the unique role of VEGF in osteoclastogenesis in RA. VEGF-induced receptor activator of nuclear factor ҡB ligand (RANKL) expression was determined in RA synovial fibroblasts by real-time PCR, luciferase assays, and ELISA. Osteoclastogenesis in peripheral blood monocytes cultured with VEGF was assessed by determining the numbers of tartrate-resistant acid phosphatase (TRAP)-positive multinucleated cells. Synovial fluid RANKL was correlated with VEGF concentration in the RA patients. VEGF stimulated the expression of RANKL in RA synovial fibroblasts. The RANKL promoter activity was upregulated by VEGF in the synovial fibroblasts transfected with RANKL-reporter plasmids. The VEGF-induced RANKL expression was decreased by the inhibition of both VEGF receptors (VEGFR) 1 and 2, Src, protein kinase C (PKC) and p38 MAPK. VEGF induced osteoclast differentiation from monocytes in the absence of RANKL and this was decreased by the inhibition of VEGFR1 and 2, Src, PKC and p38 MAPK. On coculturing with VEGF-prestimulated RA synovial fibroblasts, the monocytes differentiated into osteoclasts, and the osteoclastogenesis decreased by inhibition of Src and PKC pathways. VEGF plays dual roles on osteoclastogenesis in RA: direct induction of osteoclastogenesis from the precursors and stimulation of RANKL production in synovial fibroblasts, which is mediated by Src and PKC pathways. The axis of VEGF and RANKL could be a potential therapeutic target for RA-associated bone destruction.

## Introduction

Rheumatoid arthritis (RA) is a systemic autoimmune disease characterized by synovial inflammation and subsequent joint destruction [1, 2]. Proliferation of synovial fibroblasts and infiltration of inflammatory cells creates hypoxia in the synovium, which affects the gene expression for angiogenesis, inflammation, and cartilage degradation [3].

Angiogenesis plays an important role in the maintenance and progression of synovitis. This vascular process occurs during the early events of synovial proliferation, which promotes cartilage and bone destruction in later stages of RA. Vascular endothelial growth factor (VEGF) is the most potent angiogenic factor [4] produced by endothelial cells [5], fibroblasts [6], T cells [7], and macrophages [8]. The serum and synovial fluid VEGF concentrations are higher in RA patients than in osteoarthritis (OA) patients or normal controls, and the VEGF levels correlate with the RA disease activity, including factors such as erythrocyte sedimentation rate, C-reactive protein, tender and swollen joint counts, serum rheumatoid factor and quality of life [9–12]. In addition, VEGF has proinflammatory and anti-apoptotic roles in RA pathogenesis [13]. It induces tumor necrosis factor (TNF)-α and interleukin (IL)-6 from synovial fluid mononuclear cells of RA patients [14]. Moreover, several inflammatory molecules such as TNF-α, IL-1β, IL-6, macrophage-migration inhibitory factor, IL-17, IL-18, prostaglandin, nitric oxide, and transforming growth factor (TGF)-β promote VEGF production by synovial fibroblasts [10, 15–18]. VEGF increases the survival of synovial fibroblasts in RA, protects the apoptotic death of synovial fibroblasts, and interacts with neuropilin-1 [19].

VEGF is essential in bone homeostasis. It is physiologically involved in bone development and fracture repair. On the other hand, it plays a key role in the bone destruction process in pathological conditions such as cancer metastasis. Cartilage and bone destructions are also important processes in RA because they irreversibly disable joints. Bone destruction in RA is closely associated with bone resorption of osteoclasts in the synovial tissues, and the major inducer of osteoclasts is receptor activator of nuclear factor-kappaB ligand (RANKL) [20]. While synovial macrophages and fibroblasts express VEGF, osteoclasts and their precursors possess VEGF receptors (VEGFRs) [21].

Anti-VEGF therapies inhibiting VEGF itself or its receptors reduce the clinical severity and joint destruction in animal models of RA [22–24]. VEGF, instead of macrophage colony-stimulating factor (M-CSF), supports RANKL for osteoclast differentiation from peripheral blood monocytes [25] and increases osteoclast survival and resorptive activity [26]. However, the unique role of VEGF in bone destruction in RA is not completely clear.

In this study, we aimed to determine the role of VEGF in the bone destructive process of RA. Accordingly, we assessed the effects of VEGF on RANKL induction from synovial fibroblasts and their signaling pathways and the effects of RANKL on osteoclast differentiation from their precursors and signaling pathways.

## Materials and Methods

### Patients

Written informed consent was obtained from all patients and healthy volunteers, and the experimental protocol was approved by Institutional Review Board for Human Research, Konkuk University Hospital (KUH1010186). Synovial fluid and serum were obtained from 32 RA patients (age 51.8±1.5 years and 25 females and 7 males), who fulfilled the 1987 revised criteria of the American College of Rheumatology (ACR) [27].

### Isolation of synovial fibroblasts

Synovial tissues were isolated from eight RA patients (mean age 63.4 ± 4.6 years) who fulfilled the 1987 revised criteria of the ACR and five osteoarthritis patients (mean age 56.6±4.7 years) undergoing total knee replacement surgery, who fulfilled the ACR classification of osteoarthritis of the knee and had advanced osteoarthritis with Kellgren-Lawrens grade 4 [28, 29]. Synovial fibroblasts were isolated by enzymatic digestion of the synovial tissues, as described previously [30].

### Reagents

Recombinant human VEGF_165_, RANKL, and macrophage colony-stimulating factor (M-CSF) were purchased from R&D Systems (Minneapolis, MN). Inhibitors of signal molecules such as Src inhibitor (PP1), protein kinase C (PKC) inhibitor (PKC inhibitor peptide) and P38 mitogen-activated protein kinase (MAPK) inhibitor (SB203580) were obtained from Calbiochem (Schwalbach, Germany). Anti-human VEGF, anti-human VEGFR1, Anti-human VEGFR2 and anti-CD55 antibodies were purchased from R&D Systems.

### Confocal microscopic analysis of synovial VEGF and RANKL co-expression

Immunofluorescence staining was performed on sections of synovial tissues. Briefly, synovial tissues were obtained from patients with RA and OA. Cryosections (7 μm thick) were fixed with acetone for 15 min at room temperature, and blocked with 10% goat serum for 30 min at room temperature. After overnight incubation at 4°C with anti-human RANKL antibody (Santa Cruz Biotechnology, Santa Cruz, CA) and anti-VEGF antibody (R&D Systems), the samples were incubated with the secondary antibodies, anti-mouse FITC (Santa Cruz Biotechnology) and anti-rabbit PE (Southern Biotech, Birmingham, AL). The stained sections were visualized under a Zeiss microscope (LSM 510 Meta; Carl Zeiss, Oberkochen, Germany) at ×200 and ×400 magnifications.

### Expression of RANKL mRNA by real-time PCR

Synovial fibroblasts were stimulated with various rhVEGF concentrations. For RANKL signal pathway analysis, the FLS were incubated in the presence or absence of P38 mitogen-activated protein kinase (MAPK) inhibitor (10 nM), Src inhibitor (10 nM), PKC inhibitor (5 nM), anti-VEGFR-1, or anti-VEGFR-2 for 1 h before the addition of rhVEGF. After incubation for 72 h, mRNA was extracted using RNAzol B (Biotex Laboratories, Houston, TX) according to the manufacturer’s instructions. Reverse transcription of 2 μg of total mRNA was performed at 42°C using the Superscript™ reverse transcription system (Takara, Shiga, Japan). Polymerase chain reaction (PCR) was performed in a final volume of 20 μl in capillary tubes in a LightCycler instrument (Roche Diagnostics, Mannheim, Germany). The reaction mixture contained 2-μl LightCycler FastStart DNA MasterMix for SYBR Green I (Roche Diagnostics), 0.5 μM of each primer, 4 mM MgCl_2_, and 2 μl of template DNA. All the capillaries were amplified in a LightCycler instrument with activation of polymerase (95°C for 10 min), followed by 45 cycles of 10 s at 95°C, 10 s at 60°C (β-actin) or 59°C (RANKL), and 10 s at 72°C. The temperature transition rate was 20°C/s for all steps. The double-stranded PCR product was measured during the 72°C extension step by detection of fluorescence associated with the binding of SYBR Green I to the product. The fluorescence curves were analyzed using LightCycler software v. 3.0 (Roche Diagnostics). The LightCycler was used to quantify RANKL mRNA. The relative expression level of each sample was calculated as the level of RANKL normalized to that of the endogenously expressed housekeeping gene (β-actin). Primers were designed using Primer3 software (http://frodo.wi.mit.edu/). Primer efficiencies were determined by construction of a standard curve using 5-fold serial dilutions of pooled cDNA template. Primer specificity was determined by melt curve analysis and gel electrophoresis. The relative expression level of each sample was calculated as the level of RANKL, tartrate-resistant acid phosphatase (TRAP), RANK, calcitonin receptor (CTR), cathepsin K, or MMP-9 normalized to the endogenously expressed housekeeping gene for beta-actin. The following primers were used for each molecule: for RANKL, 5’-ACC AGC ATC AAA ATC CCA AG-3’ (sense) and 5’-CCC CAA AGT ATG TTG CAT CC-3’ (antisense); for TRAP, 5’-GAC CAC CTT GGC AAT GTC TCT G-3’ (sense) and 5’-TGG CTG AGG AAG TCA TCT GAG TTG-3’ (antisense); for Cathepsin K, 5’-TGA GGC TTC TCT TGG TGT CCA TAC-3’ (sense) and 5’-AAA GGG TGT CAT TAC TGC GGG-3’ (antisense); for calcitonin receptor, 5’-TGG TGC CAA CCA CTA TCC ATG C-3’ (sense) and 5’-CAC AAG TGC CGC CAT GAC AG-3’ (antisense); for MMP-9, 5’- CGC AGA CAT CGT CAT CCA GT-3’ (sense) and 5’- GGA TTG GCC TTG GAA GAT GA-3’(antisense); beta-actin, 5’-GGA CTT CGA GCA AGA GAT GG-3’ (sense) and 5’-TGT GTT GGC GAT CAG GTC TTT- G-3’. Melting curve analysis was performed immediately after the amplification protocol under the following conditions: 0 s (hold time) at 95°C, 15 s at 71°C and 0 s (hold time) at 95°C. The rate of temperature change was 20°C/s, except for the final step, during which it was 0.1°C/s. The melting peak generated represented the quantity of specific amplified product. The crossing point (*C*_p_) was defined as the maximum of the second derivative from the fluorescence curve. Negative controls were also included and contained all the elements of the reaction mixture except for the template DNA. All samples were processed in duplicate.

### Immunohistochemical analysis of RA synovial fibroblasts

Intracellular expression of RANKL was also increased by VEGF stimulation in the cultured RA synovial fibroblasts, as shown by in vitro cellular immunostaining 72 hours after stimulation. Immunohistochemical staining for RANKL was performed in the cultured RA synovial fibroblasts. The cultured RA synovial fibroblasts were fixed with 10% formaldehyde solution overnight at 4°C and washing. The cells were depleted of endogenous peroxidase activity by adding methanolic H2O2 and blocked with normal serum for 30 minutes. After overnight incubation at 4°C with polyclonal anti-human RANKL antibody (Santa Cruz Biotechnology) (R&D Systems), the cells were incubated for 20 minutes with the secondary antibody, biotinylated anti-rabbit IgG, and incubated with streptavidin–peroxidase complex (Vector) for 1 hour, followed by incubation with 3,3_-diaminobenzidine (Dako) for 5 minutes. The sections were counterstained with hematoxylin. The cells were photographed using an Olympus photomicroscope

### Dual luciferase assay

RANKL activity was assessed by measurements of luciferase expression in RA FLS with transient transfection of appropriate cDNA constructs and luciferase reporter vectors, namely pGL3-704 to +111. Another reporter plasmid, *Renilla* luciferase (pRLTk, Promega), was co-transfected as an internal transfection control. RANKL transcription was evaluated with pGL3-RANKL-luc plasmid containing the luciferase gene under the control of the RANKL-inducible promoter. Transient transfection of synovial fibroblasts was performed using fugene HD (Promega, Wisconsin). Briefly, cells were seeded in six-well plates at 2×10^5^ cells/well 1 day before transfection. Transfection was carried out according to the manufacturer’s recommendations using 1 μg of pGL3-RANKL reporter plasmids and 1 μg of pRLTk control plasmid. At 24 h after transfection, cells were stimulated or left unstimulated for 24 h. After 24 h, cells were lysed by 500 μL of 1× Passive Lysis Buffer (Promega). Luciferase assays were performed using the dual luciferase reporter assay kit (Promega).

### Enzyme-linked immunosorbent assay of sRANKL and VEGF

Briefly, a 96-well plate (Nunc) was coated overnight with 4 μg/ml monoclonal antibodies against soluble RANKL (sRANKL) and VEGF (R&D Systems) at 4°C. After blocking with phosphate-buffered saline (PBS)/1% bovine serum albumin (BSA)/0.05% Tween 20 for 2 h at room temperature (22–25°C), the test samples and standard recombinant sRANKL and VEGF were added to the 96-well plate and incubated at room temperature for 2 h. The plates were washed four times with PBS/Tween 20, and then incubated with 500 ng/ml biotinylated mouse monoclonal antibodies against sRANKL and VEGF for 2 h at room temperature. After washing, streptavidin–alkaline phosphate–horseradish peroxidase conjugate (Sigma) was added and the plate was incubated for 2 h, after which the plate was washed again and incubated with 1 mg/ml *p*-nitrophenyl phosphate (Sigma) dissolved in diethanolamine (Sigma) to develop the color reaction. The reaction was quenched by the addition of 1 M NaOH, and the optical density of each well was read at 405 nm. The lower limit of sRANKL and VEGF detection was 10 pg/ml. Recombinant human sRANKL and VEGF diluted in the culture medium were used as the calibration standard, whose concentrations ranged from 10 to 2000 pg/ml. A standard curve was drawn by plotting the optical density as a function of the log of the concentration of recombinant cytokines, and was used to calculate the sRANKL and VEGF concentrations in the test samples.

### Western blotting analysis

Synovial fibroblasts were incubated with various doses of rhVEGF for 1 h, and then whole-cell lysates were prepared from approximately 2×10^5^ cells by homogenization in lysis buffer and centrifuged at 14000 rpm for 15 min. The protein concentration in the supernatant was measured using the Bradford method (Bio-Rad, Hercules, CA). Protein samples were resolved by 10% sodium dodecyl sulfate–polyacrylamide electrophoresis (SDS–PAGE), and transferred onto nitrocellulose membranes (Amersham Pharmacia Biotech, Uppsala, Sweden). For western hybridization, the membranes were preincubated with 0.5% skim milk in TTBS (0.1% Tween 20 in Tris-buffered saline) at room temperature for 2 h. The primary antibody to phospho-Src, or phospho-PKC (Cell Signaling Technology Inc., Danvers, MA) was diluted 1:1000 in TTBS containing 5% bovine serum albumin (BSA), and incubated overnight at 4°C. The membranes were washed four times with TTBS, horseradish peroxidase-conjugated secondary antibody was added, and the membranes were incubated for 1 h at room temperature. After washing with TTBS, the hybridized bands were detected using an ECL detection kit and Hyperfilm-ECL reagents (Amersham Pharmacia).

### Monocyte isolation and osteoclast differentiation

Peripheral blood mononuclear cells (PBMC) were separated by Ficoll–Hypaque (Sigma Chemicals, Poole, Dorset, UK) density gradient centrifugation from the buffy coats obtained from RA patients and healthy volunteers. The cells were washed three times with sterile PBS and resuspended in RPMI 1640 (Life Technologies, Grand Island, NY) supplemented with 10% fetal bovine serum (FBS), 2 mM l-glutamine, and 1% penicillin–streptomycin (complete medium). Freshly isolated PBMC were incubated at 37°C in complete medium and allowed to adhere for 45 min. The non-adherent cells were removed and the adherent cells were washed with sterile PBS, harvested with a rubber policeman, and stained with monocyte-specific anti-CD14 monoclonal antibody to assess the purity of the preparation. Ninety percent of the isolated cells expressed CD14. The osteoclast precursors were prepared using the monocyte-enriched fraction from the peripheral blood.

RA synovial fibroblasts were pretreated with 20 ng/ml rhVEGF for 3 days, after which monocytes were added to each well along with different signal inhibitors. As described above, the isolated human monocytes (5×10^4^ cells/well) were added to the VEGF-pretreated FLS with fresh media. The cells were cocultured for 3 weeks in α-minimal essential medium (MEM) and 10% heat-inactivated FBS in the presence of 25 ng/ml rhM-CSF. The medium was changed on day 3 and then every other day thereafter. The addition of rhRANKL protein, prepared as described previously [31], was used as a positive control. On day 21, tartrate-resistant acid phosphatase (TRAP)-positive cells were identified using a leukocyte acid phosphatase kit according to the manufacturer’s protocol (Sigma–Aldrich, St. Louis, MO) [32].

### Statistical analysis

Results are expressed as the mean ± S.E.M. Statistical differences were analyzed using the Mann–Whitney *U* test or one-way analysis of variance (ANOVA) with Tukey’s multiple comparison post-hoc test. The correlation was analyzed using Pearson’s correlation coefficient. A *P* value < 0.05 was considered statistically significant.

## Results

### Expression of VEGF and RANKL in synovial fluids and synovial tissues of RA patients

**Fig 1:**
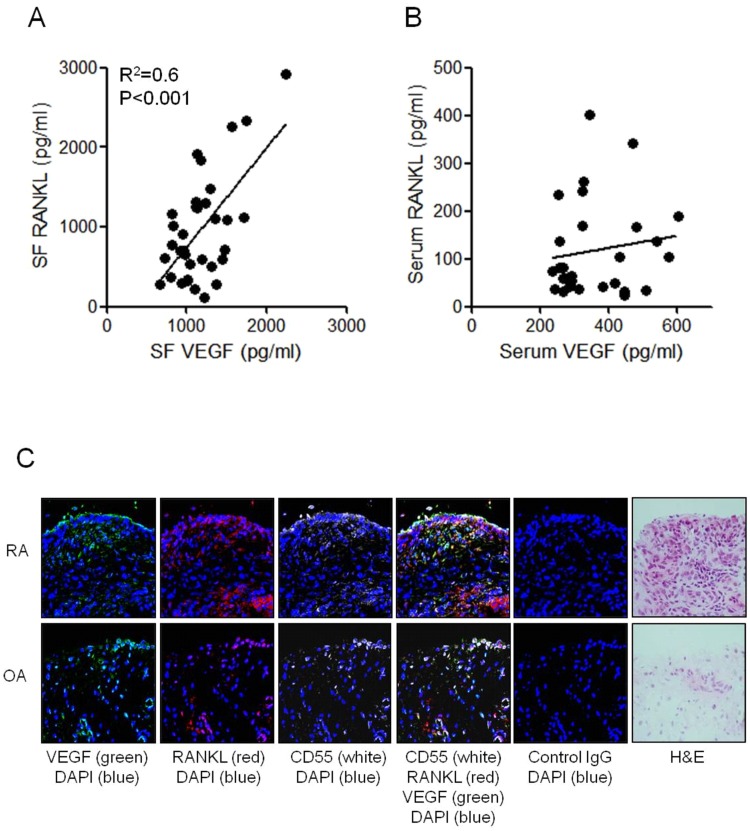
The expression of VEGF and RANKL in the synovial fluid, serum, and synovial tissues of RA patients. **(A)** The synovial fluid samples of 32 RA patients were collected and their VEGF and soluble RANKL concentrations were determined by sandwich ELISA. **(B)** The serum samples of 32 RA patients were collected and their VEGF and soluble RANKL concentrations were determined by sandwich ELISA. Each dot expresses the results from an individual patient. **(C)** The synovial tissues of patients with RA and osteoarthritis (OA) were simultaneously labeled with anti-VEGF (green), anti-RANKL (red), and CD55 (white) antibodies and then photographed under appropriate filters. The merged image shows co-localization of the three markers (yellow). Sections were counterstained with DAPI staining. The figures are representative of three independent experiments (original magnification 400×).

Both VEGF and RANKL were overexpressed in the serum, synovial fluid, and synovial tissues of RA patients [9, 33]. A significant positive correlation was noted between VEGF and RANKL concentrations in the synovial fluid of 32 RA patients (r^2^ = 0.6, *P* < 0.001) (Fig 1A), while no such relationship was noted between their respective serum concentrations (Fig 1B). The expressions of VEGF and RANKL in RA and OA synovial tissues were determined by confocal microscopy with multiple-fluorescence staining. VEGF, RANKL, and CD55 were abundantly expressed in the lining and sub-lining area of RA synovium, but negligibly expressed in the OA synovium. By triple immunofluorescent labeling for CD55, VEGF, and RANKL, the RANKL expression consistently overlapped with the expressions of VEGF and CD55 (Fig 1C). These results showed that CD55-positive synovial fibroblasts express both VEGF and RANKL.

### VEGF-induced RANKL expression in RA synovial fibroblasts

**Fig 2:**
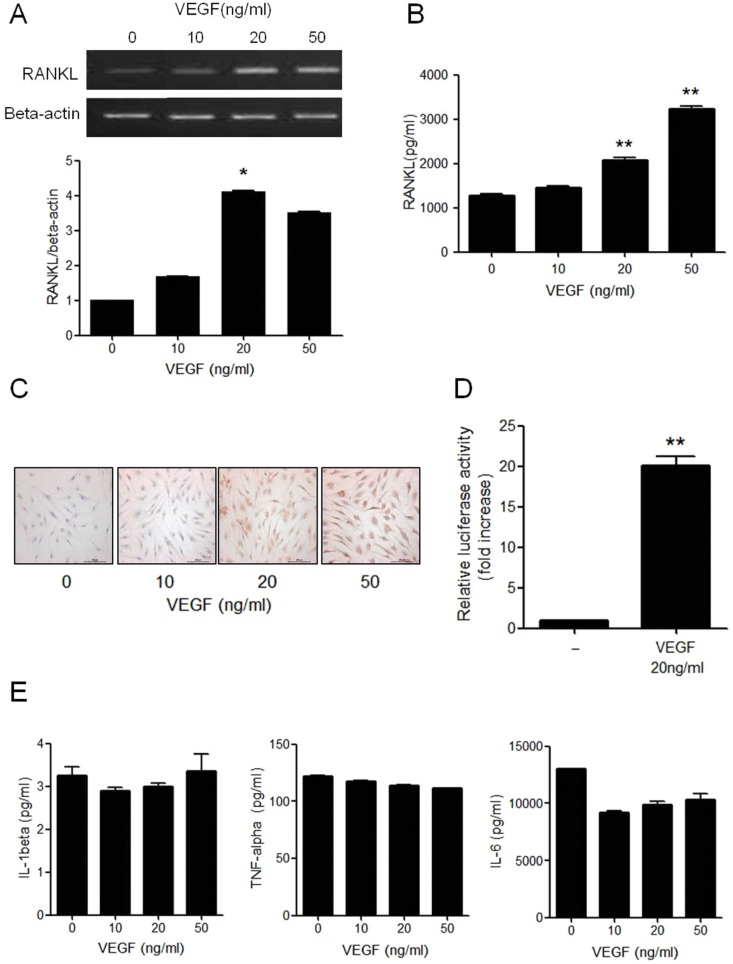
VEGF-induced RANKL expression in RA synovial fibroblasts. **(A)** After RA synovial fibroblasts were cultured with 0–50 ng/ml of VEGF for 72 h, the RANKL mRNA expression determined by RT-PCR. Data were normalized to beta-actin and reported in relative expression units. The figure is representative of three experiments. **(B)** RA synovial fibroblasts were cultured with VEGF for 72 h, and RANKL concentration in the cultured media was measured by sandwich ELISA. **(C)** RA synovial fibroblasts were cultured with VEGF for 72 h and then stained with anti-RANKL antibodies (red) (original magnification 400×). The figures are representative of three independent experiments. **(D)** Triplicate wells of RA synovial fibroblasts were transfected with 1 μg of pGL3-RANKL reporter plasmids and 1 μg of pRLTk control plasmid. Both firefly and renilla luminescence were measured after 24 h incubation with 20ng/ml of VEGF. **(E)** After RA synovial fibroblasts were cultured with VEGF for 72 h, the concentrations of IL-1β, TNF-α, and IL-6 in the cultured media was determined by sandwich ELISA. The data represent the mean ± SEM of three independent experiments. *P < 0.05, **P < 0.01.

After stimulation of RA synovial fibroblasts with VEGF, the expression of RANKL mRNA was determined by RT-PCR. The expression of RANKL mRNA was increased in a dose-dependent manner, with maximal effect exerted by 20 ng/ml VEGF (Fig 2A). *In vitro* cellular immunostaining 72 h after VEGF stimulation showed that the production of RANKL protein was increased in the cultured media (Fig 2B) and that the intracellular RANKL expression was increased in the cultured synovial fibroblasts (Fig 2C). The experimental doses of VEGF had no cytotoxic or proliferative effects on the synovial fibroblasts (data not shown). Protein interaction between VEGF and RANKL was assessed by the pGL3-luciferase reporter gene assay. The RANKL activity was increased by 20-fold in the VEGF-stimulated condition than in the un-stimulated condition transfected with pGL3-RANKL Luc and Renilla Luc (Fig 2D). To determine whether the VEGF-induced RANKL expression in synovial fibroblasts was mediated by proinflammatory cytokines, we assessed the production of proinflammatory cytokines after VEGF stimulation. We found that the stimulation of synovial fibroblasts with VEGF did not change the production of IL-1β, IL-6, and TNF-α in the cultured media (Fig 2E).

### Intracellular signaling pathways in VEGF-induced RANKL expression

**Fig 3:**
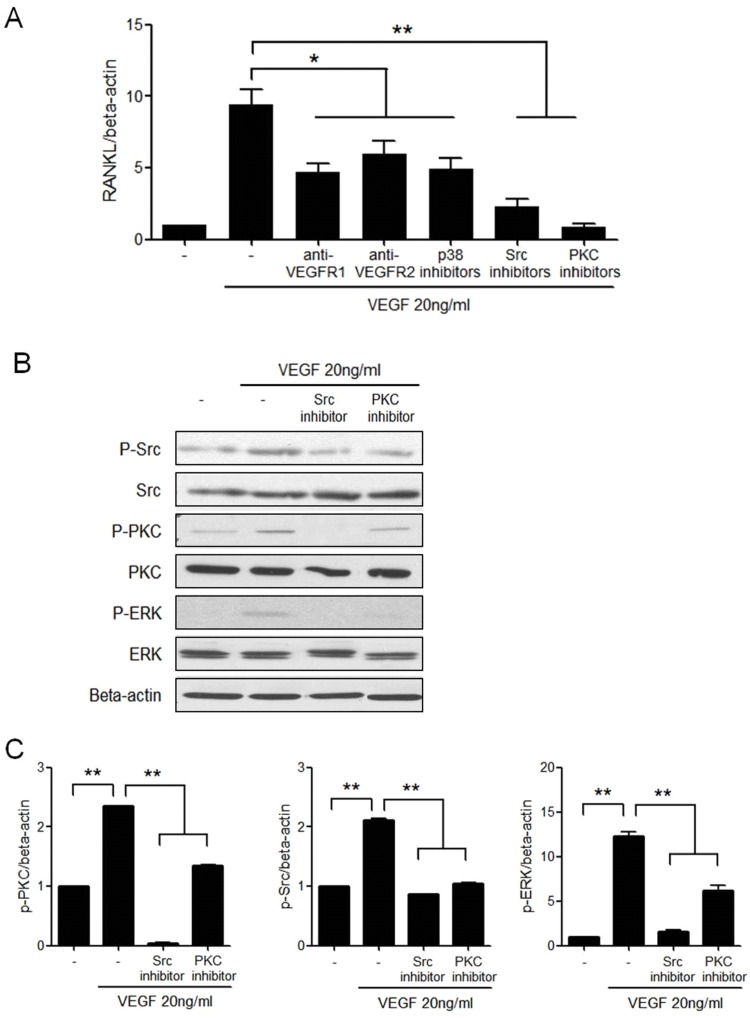
The signaling pathways involved in the VEGF-induced RANKL expression in RA synovial fibroblasts. **(A)** RA synovial fibroblasts were pretreated with anti-VEGFR1 (20 ng/ml), anti-VEGFR2 (20 ng/ml), SB203580, a p38 MAPK inhibitor (10 nM), Src inhibitor (10 nM), or PKC inhibitor (5 nM) for 1 h, and then cultured with 20 ng/ml VEGF for 72 h. The expression of RANKL mRNA was determined by real time-PCR. Data were normalized to beta-actin and reported in relative expression units. **(B)** RA synovial fibroblasts were stimulated with 20 ng/ml VEGF, the phosphorylated forms of Src, PKC, and ERK were detected by western blotting. The figures are representative of three independent experiments. **(C)** Stimulation of RA synovial fibroblasts with VEGF activated the phosphorylation of p-Src, Src, p-PKC, PKC, p-ERK and ERK as detected by Western blotting and shown by the ratio of phosphorylated to total proteins. Data were normalized to beta-actin and reported in relative expression units. The figure represents one of three independent experiments. The data represent the mean ± SEM of three independent experiments. *P < 0.05, **P < 0.01.

Synovial fibroblasts express VEGFRs, which in turn produce RANKL. Therefore, we investigated the signaling pathways involved in the VEGF-induced RANKL production in synovial fibroblasts. There are two kinds of VEGFRs—VEGFR1 and VEGFR2 [34]. To identify the intracellular signaling pathways mediating VEGF-induced RANKL expression, RA synovial fibroblasts were preincubated with anti-VEGFRs or signal inhibitors for 1 h and then cultured with VEGF for 72 h. The expression of RANKL mRNA was decreased significantly after inhibition of the activities of VEGFR1, VEGFR2, p38 MAPK, Src, and PKC (P < 0.05 for VEGFR1, VEGFR2, and p38 MAPK; P < 0.005 for Src and PKC) (Fig 3A). No cytotoxic effect of the inhibitors was observed on the synovial fibroblasts under the experimental concentrations (data not shown). Western blotting revealed that VEGF induced the phosphorylation of Src, PKC, and ERK in synovial fibroblasts (P < 0.005) (Fig 3B and 3C).

### VEGF-induced osteoclast differentiation from peripheral blood monocytes

**Fig 4:**
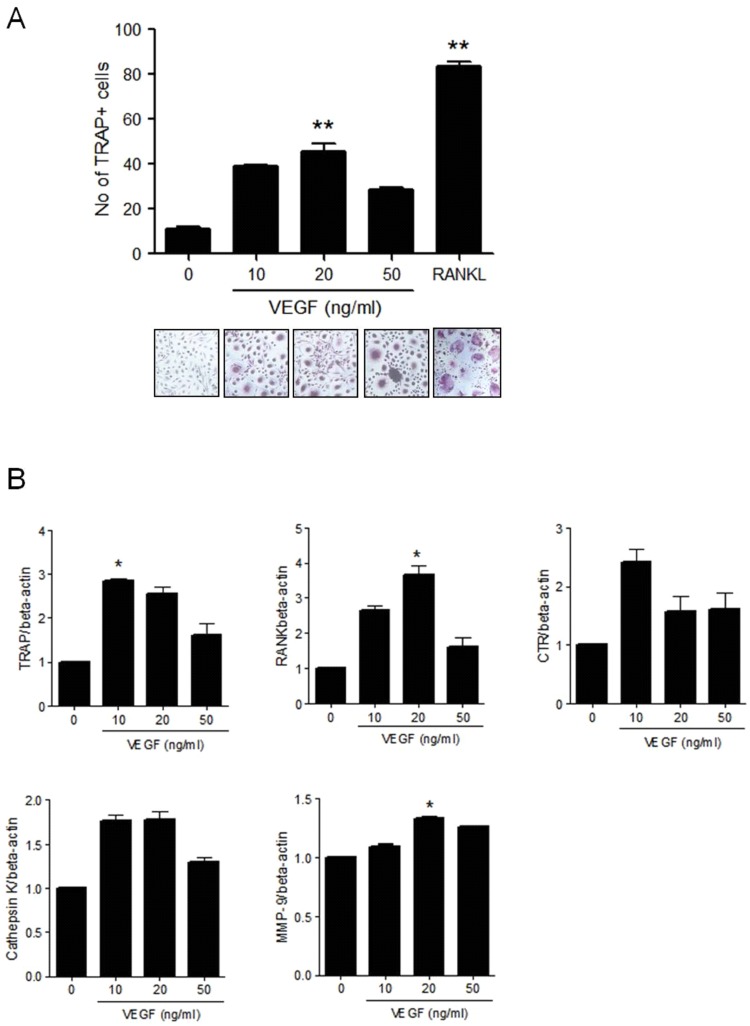
VEGF-induced osteoclast differentiation from CD14+ monocytes isolated from peripheral blood. **(A)** CD14+ monocytes isolated from peripheral blood of RA patients were cultured with 25 ng/ml M-CSF and 0–50 ng/ml VEGF or 30 ng/ml RANKL. After maximal 21 days of culturing, TRAP-positive multinucleated cells were counted. The figures represent one of three independent experiments. **(B)** The gene expression of osteoclast markers such as TRAP, RANK, CTR, cathepsin K, and MMP-9 from differentiated osteoclasts measured by real-time PCR. Data were normalized to beta-actin and reported in relative expression units. The data represent the mean ± SEM of three independent experiments. *P < 0.05, **P < 0.01.

Peripheral blood monocytes can differentiate into TRAP+ multinucleated osteoclasts in the presence of RANKL and M-CSF [35–37]. Monocytes and macrophages also have VEGFRs, and VEGFR1 is the major receptor for monocyte migration and osteoclastogenesis [38, 39]. To evaluate the direct effect of VEGF on the induction of osteoclastogenesis, isolated CD14+ monocytes from the peripheral blood of RA patients were cultured with VEGF and M-CSF in the absence of RANKL. After 21 days of culturing, TRAP+ multinucleated osteoclasts were differentiated from the monocytes in a dose-dependent manner, with maximal effect exerted by 20 ng/ml VEGF (p<0.005). However, the osteoclastogenic effect of VEGF was smaller than that of RANKL owing to lesser cell numbers and smaller cell sizes of the differentiated osteoclasts (Fig 4A). The expressions of osteoclast makers such as TRAP, RANK, and MMP-9 were significantly increased by VEGF stimulation (P < 0.05) (Fig 4B).

### Intracellular signaling pathways for VEGF-induced osteoclastogenesis

**Fig 5:**
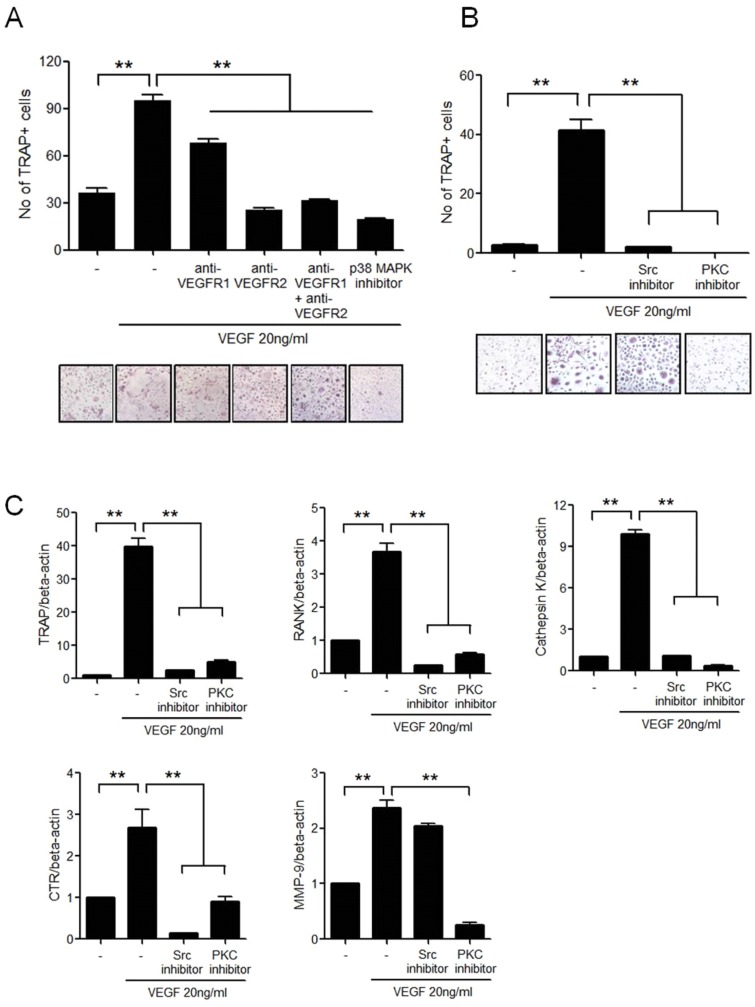
The signaling pathways for VEGF-induced osteoclast differentiation from peripheral blood monocytes. **(A)** CD14+ monocytes were cultured with M-CSF and 20 ng/ml of VEGF in the presence of 20ng/ml of anti-VEGFR1, 20ng/ml of anti-VEGFR2, or 10nM of p38 MAPK inhibitor. After 21 days of culturing, TRAP+ multinucleated cells were counted. The figure represents one of three independent experiments. **(B)** CD14+ monocytes were cultured with M-CSF and 20 ng/ml of VEGF in the presence of Src inhibitor (10 nM), or PKC inhibitor (5 nM). After 21 days of culturing, TRAP+ multinucleated cells were counted. The figure represents one of three independent experiments. **(C)** The gene expression of TRAP, RANK, CTR, cathepsin K, and MMP-9 from differentiated osteoclasts was measured by real-time PCR. Data were normalized to beta-actin and reported in relative expression units. The data represent the mean ± SEM of three independent experiments. *P < 0.05, **P < 0.01.

To assess the intracellular signaling pathways mediating VEGF-induced osteoclast differentiation, the isolated monocytes from RA patients were cultured with VEGF, M-CSF, and different signal inhibitors. The differentiation of the monocytes into TRAP+ osteoclasts was significantly decreased due to inhibition of VEGFR1, VEGFR2, p38 MAPK, Src, and PKC; however, the inhibition of VEGFR1 showed partial effect while the inhibition of VEGFR2 p38 MAPK, Src, and PKC completely reduced the osteoclastogenesis (Fig 5A and 5B). The expressions of differentiated osteoclast markers such as RANK, TRAP, CTR, cathepsin K, and MMP-9 mRNA were also decreased by inhibition of Src and PKC (Fig 5C).

### Osteoclastogenesis from the peripheral blood monocytes cultured with VEGF-prestimulated RA synovial fibroblasts

**Fig 6:**
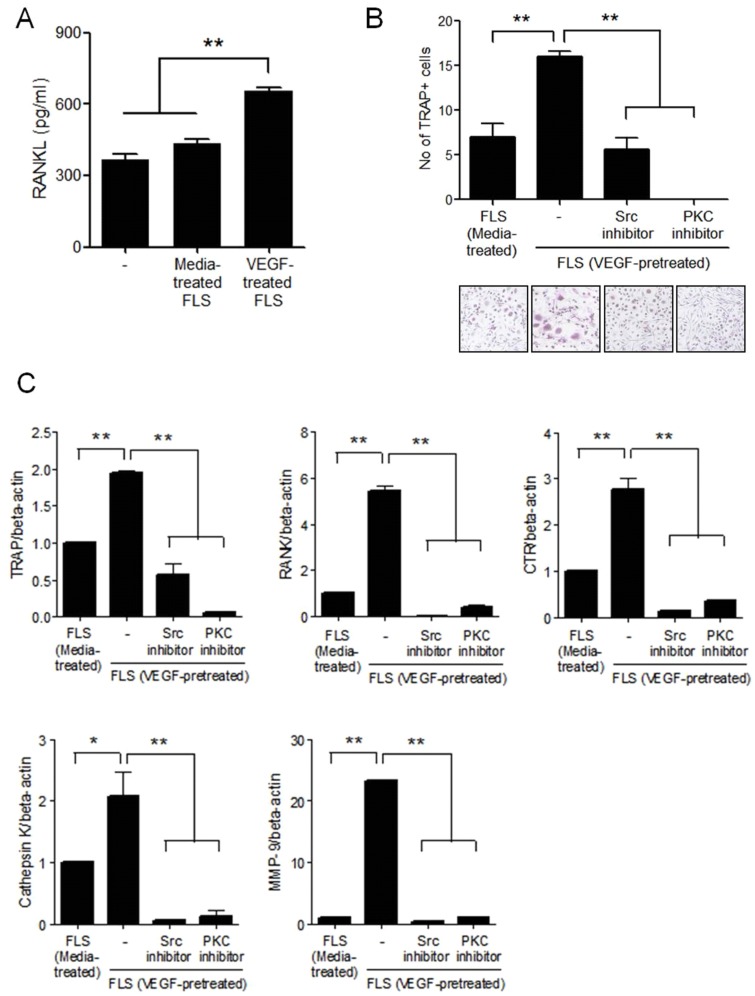
Induction of osteoclastogenesis by VEGF-pretreated RA synovial fibroblasts. **(A)** RA synovial fibroblasts were cultured with 20 ng/ml of VEGF for 72 h and RANKL production was quantified using ELISA in the cultured media. **(B)** RA synovial fibroblasts were preincubated with 20 ng/ml of VEGF for 72 h and Src inhibitor (10 nM) or PKC inhibitor (5 nM) and then cocultured with CD14+ monocytes from the peripheral blood in the presence of M-CSF. After 21 days of culturing, TRAP-positive multinucleated cells were counted. The figure represents one of three independent experiments. **(B)** The gene expressions of TRAP, RANK, CTR, cathepsin K, and MMP-9 from differentiated osteoclasts measured by real-time PCR. Data were normalized to beta-actin and reported in relative expression units. The data represent the mean ± SEM of three independent experiments. *P < 0.05, **P < 0.01.

Synovial fibroblasts cultured with peripheral blood monocyte can induce osteoclast differentiation in cell culture. In an actual disease situation, the RA synovial fibroblasts are already affected by inflammatory molecules. To determine the effects of VEGF on osteoclastogenesis indirectly, RA synovial fibroblasts were pretreated with VEGF and then cultured with peripheral blood monocytes in the presence of M-CSF. VEGF-pretreated synovial fibroblasts produced more RANKL than non-treated synovial fibroblasts (Fig 6A). When CD14+ monocytes were cocultured with VEGF-pretreated synovial fibroblasts in the absence of RANKL, differentiation into TRAP+ multinucleated cells was greater than monocytes cocultured with untreated synovial fibroblasts (Fig 6B). The osteoclast differentiation was significantly decreased after inhibition of Src and PKC signaling (Fig 6B). The expressions of differentiated osteoclast markers such as RANK, TRAP, CTR, cathepsin K, and MMP-9 mRNA were also decreased due to inhibition of Src and PKC signaling (Fig 6C).

## Discussion

VEGF is a dimeric glycoprotein that induces the proliferation and migration of endothelial cells to form new blood vessels and increases the vascular permeability [40]. VEGF is upregulated in synovial macrophages and synovial fibroblasts of RA patients, and cultured synovial cells can produce VEGF under hypoxia or with stimulation by IL-1, IL-6, IL-17, IL-18, prostaglandin, TGF-*β*, or CD40 ligation [10, 16, 41, 42]. Furthermore, VEGF-deficiency has been reported to reduce synovial inflammation and angiogenesis in antigen-induced mice models of arthritis [22]. In addition to its role in angiogenesis, VEGF increases the production of proinflammatory cytokines such as TNF-*α* and IL-6 by peripheral blood and synovial fluid mononuclear cells of RA patients. VEGF also prevents apoptosis of synovial fibroblasts and promotes chemokine productions by endothelial cells, such as MCP-1 and IL-8 [43, 44]. TNF-*α* and IL-6, in turn, further enhance the capabilities of macrophages and synoviocytes to secrete VEGF and stimulate endothelial cells to induce cell contact-mediated macrophage activation, which generates a positive feedback loop. Thus, VEGF serves as a functional bridge between angiogenesis and inflammation.

In order to determine the role of VEGF on the bone destruction process of RA, we targeted RANKL and osteoclasts as they are the major molecules and cells for RA-associated bone destruction. As RANKL is the major mediator of osteoclast differentiation and activation, we also analyzed the relationship between RANKL and VEGF concentrations in the serum and synovial fluid of RA patients. We found a strong correlation between RANKL and VEGF concentrations in the synovial fluid, which suggests a pathogenic relationship between these molecules.

Next, we investigated the effect of VEGF on the induction of RANKL in RA synovial fibroblasts. We found that VEGF activated synovial fibroblasts to express and produce RANKL, which indicates that VEGF creates RANKL-rich synovial environment for promoting osteoclast differentiation from synovial osteoclast precursors such as macrophage-like synoviocytes and monocytes/macrophages in the synovial fluid. TNF-α and IL-1β are other cytokines that are involved in osteoclastogenesis of RA [45, 46]. However, VEGF did not affect the production of these cytokines in the synovial fibroblast culture, suggesting that VEGF independently increases the production of RANKL in the synovial fibroblasts. Confocal staining revealed that synovial fibroblasts also produce VEGF. Thus, both VEGF and RANKL can be produced via autocrine and paracrine manners to create a vicious cycle of angiogenesis and bone destruction.

To determine the effect of VEGF on osteoclastogenesis, peripheral blood CD14+ monocytes were isolated and cultured with VEGF and M-CSF in the absence of RANKL. RANKL is known to be essential for the induction of osteoclastogenesis in peripheral blood monocytes. However, in this study, we found that VEGF and M-CSF induced osteoclast differentiation from monocytes independent of RANKL. This result suggests that VEGF can substitute RANKL in the induction of osteoclastogenesis. The degree of osteoclast differentiation in cultures with VEGF was lower in the absence of RANKL than in the presence of RANKL, which is the most powerful inducer for osteoclastogenesis. Our culture system of VEGF and M-CSF without RANKL can elucidate the direct osteoclastogenic effect of VEGF. In previous studies, we found that macrophage-migration inhibitory factor, IL-22, and stromal cell derived factor-1 directly induce osteoclastogenesis in the presence of M-CSF, substituting for RANKL [18, 33, 47]. We believe that, in pathologically osteoclastogenic conditions like RA, various inflammatory molecules other than RANKL can induce osteoclastogenesis, while RANKL is essential for osteoclastogenesis in normal bone development and maintenance.

VEGF interacts with two kinds of receptors—VEGFR-1 [also known as Fms-like tyrosine kinase-1 (Flt-1)] and VEGFR-2 [also known as fetal liver kinase-1 (Flk-1) or kinase insert domain-containing receptor (KDR)]. VEGFR1 is involved in the migration of endothelial cells and monocytes/macrophages as well as in angiogenesis associated with cancer and inflammation mainly via the p38 MAPK signaling pathway. VEGFR2 is mainly associated with endothelial cell growth, cell survival, and vascular permeability mediated mainly via the Src and PKC signaling pathways [34, 39]. RA synovial cells contain both VEGFR1 and 2 [24, 48]. VEGFR1 is constitutively expressed in both RA and OA synovial tissues, while VEGFR2 is abundantly expressed in the RA synovial tissues, but undetectable in OA synovial tissues [48, 49]. Moreover, VEGFR2 is closely associated with VEGF_165_ [48]. In this study, RANKL expression of synovial fibroblasts and VEGF-induced osteoclast differentiation were decreased by blockage of both VEGFR1 and VEGFR2. Intracellular signals below VEGFRs, Src, PKC and p38 MAPK pathways were mainly involved. When osteoclast precursors are cultured for differentiation into osteoclasts, the expression of VEGFR2 genes increases, but that of VEGFR1 or neuropilin-1 decreases [26]; moreover, RAF265, a VEGFR2 inhibitor, prevents osteoclast formation and retards resorptive capacity of the peripheral blood mononuclear cells [50]. The results of these previous studies are consistent with our results, suggesting that VEGF-induced osteoclastogenesis is mediated by VEGFR-2. On the contrary, another study showed that the activation of VEGFR1 in the presence of RANKL induces osteoclast differentiation [25]. In the animal models of RA, such as collagen-induced arthritis and K/BxN models, the blockage of VEGFR1 and not of VEGFR2 suppresses joint destruction and exerts therapeutic effects [23, 24]. Bone destruction in RA is the final outcome of combined activation of synovial and inflammatory cells, osteoclasts, angiogenesis, migration, and cartilage loss, that is, various molecules and receptors are involved in this process. Further detailed research is required to investigate the direct linkage between VEGF and osteoclastogenesis.

We also investigated whether VEGF-pretreated synovial fibroblasts affect osteoclast differentiation from precursors. This coculture system inclusive of VEGF-pretreated synovial fibroblasts and osteoclast precursors reflects the actual clinical condition of affected joints, because interactive networks exist among several cytokines and synovial cells in the RA synovium. When RA synovial fibroblasts were pretreated with VEGF and then cocultured with CD14+ monocytes, TRAP+ cells were generated in the absence of RANKL. This finding indicates that VEGF has both a direct effect on osteoclastogenesis and an indirect effect through stimulation of synovial fibroblasts, which can produce RANKL and stimulate osteoclast precursors.

There are some limitations in this study. First, this study examined in vitro mechanism of VEGF-induced osteoclastogenesis in the cellular and molecular levels, so there is possibility of inconsistency with clinical phenomenon in RA joints. Second, the differentiated osteoclasts were counted without study for their bone resorbing function. Third, clinical significance of the relationship between VEGF and RANKL or osteoclastogenesis was not determined because our samples had little clinical information of RA patients. Forth, VEGFR1 and 2 were studied for signal pathways involved in VEGF-induced osteoclastogenesis, but the signal of VEGFR3 was not determined.

In conclusion, VEGF stimulated synovial fibroblasts to produce RANKL and thus indirectly induced osteoclastogenesis in the RA synovium. VEGF also induced osteoclastogenesis directly from the peripheral blood monocytes. Thus, the dual osteoclastogenic effects of VEGF are mediated by Src/PKC and p38 MAPK signaling pathways.
